# A new species of *Huffmanela* (Nematoda: Trichosomoididae) parasitizing *Colomesus psittacus* (Tetraodontiformes) from Marajó Island, Pará State, Brazil

**DOI:** 10.1590/S1984-29612022045

**Published:** 2022-08-22

**Authors:** Elaine Lopes de Carvalho, Ricardo Luis Sousa Santana, Gilson Campos Corrêa, José Ledamir Sindeaux, Raul Henrique da Silva Pinheiro, Elane Guerreiro Giese

**Affiliations:** 1 Programa de Pós-graduação em Saúde e Produção Animal na Amazônia, Instituto da Saúde e Produção Animal, Universidade Federal Rural da Amazônia – UFRA, Belém, PA, Brasil; 2 Laboratório de Histologia e Embriologia Animal, Instituto da Saúde e Produção Animal, Universidade Federal Rural da Amazônia – UFRA, Belém, PA, Brasil; 3 Universidade Federal Rural da Amazônia – UFRA, Belém, PA, Brasil; 4 Casa-Escola da Pesca, Fundação Centro de Referência em Educação Ambiental Escola Bosque Professor Eidorfe Moreira – FUNBOSQUE, Belém, PA, Brasil

**Keywords:** Trichosomoididae, Huffmanela, Pisces, Tetraodontiformes, Amazon, Trichosomoididae, Huffmanela, Pisces, Tetraodontiformes, Amazônia

## Abstract

The puffer fish *Colomesus psittacus*, is not commercialized on Marajó Island. They are captured as bycacth and discarded dead in the environment in artisanal fisheries that occur in the estuaries of northern Brazil. In this sense, the objective was to identify the parasites present in the gills and to evaluate the histopathological alterations caused by these nematodes of the genus *Huffmanela*. Fifty-five fish were analyzed, and thirty-five specimens showed the parasite in the gills. Morphological characteristics suggest that it is a new species of the genus *Huffmanela,* and the histopathological exams showed an edematous inflammation in the secondary lamella and the presence of eggs of this nematode, which is the first record of this parasite in *C. psittacus* in Brazil.

## Resumo

O baiacu amazônico *Colomesus psittacus* é um peixe de importância comercial para a aquariofilia, além de ser capturado como *bycacth* e descartado morto no ambiente nas pescarias artesanais que ocorrem nos estuários da região Norte do Brasil. Nesse sentido, objetivou-se identificar e descrever nematódeos do gênero *Huffmanela* em baiacus oriundos da ilha de Marajó, bem como as alterações histopatológicas causadas por eles. Cinquenta e cinco peixes foram analisados, e trinta e cinco espécimes apresentaram o parasita nas branquias. Características morfológicas sugerem tratar-se de uma nova espécie do gênero *Huffmanela*, e os exames histopatológicos demonstraram uma inflamação edematosa na lamela secundária e presença de ovos desse nematódeo. Esse fato se torna o primeiro registro desse gênero parasito em *C. psittacus* no Brasil.

## Introduction

The puffer fish *Colomesus psittacus* (Bloch & Schneider, 1801) is often found in freshwater demersal environments but can also inhabit estuarine or marine waters. It has a distribution from Venezuela to the center north of Brazil, including the state of Pará (Brazil) (Cervigon et al., 1992; Camargo & Maia, 2008). Their food source consists of mollusks and other small benthic organisms (Szpilman, 2002).

Puffer fish, as they are popularly known, are still usually captured as bycatch and discarded dead in the environment in high densities by artisanal fisheries that occur in estuaries in northern Brazil (Barros et al., 2011; Fonseca & Souza, 2013). The species *C. psittacus* is also used as bait in fishing for *Epinephelus itajara* according to Pereira et al. (2020), and for *Brachyplatystoma filamentosum* and *Sciades parkeri* around Marajó Island. However, the species is not of interest as a protein source in northern Brazil because of its toxicity (Krumme et al., 2007).

Due to the importance of this species, knowledge of its parasitic ecology is of fundamental importance. Some previous studies with helminths in *C. psittacus* have been carried out and have identified the presence of *Rohdella* sp. (Silva et al., 2013); *Rohdella amazonica* (Giese et al., 2015), *Gnathostoma* sp. and *Cucullanus marajoara* (Pinheiro et al., 2017, 2018). All of these were intestinal parasites.

Thus, this study sought to taxonomically identify specimens of Trichosomoididae nematodes, and the lesions caused by these parasites found in the gills of *C. psittacus* on Marajó Island, Pará.

## Material and Methods

In the year 2021, fifty-five specimens of *C. psittacus* were acquired in fishing pens located in the municipality of Soure (00º 43' 00” S; 48º 31' 24” W), Marajó Island, State of Pará. The fish in the region are not used for meat consumption or sale in local shops and the specimens were transported to the laboratory cooled, then the gills were separated in Petri dishes containing 0.9% NaCl saline solution and analyzed under a stereomicroscope (Leica ES2) for the search for helminths.

After verifying the presence of nematodes in the gills, cleaned in physiological solution, they were fixed in A.F.A solution (93 parts of 70% ethyl alcohol, 5 parts of formaldehyde and 2 parts of glacial acetic acid) for morphological and morphometric analyses. Thirty males and females were clarified with Lactophenol Aman (0.5%), observed under light microscopy and photographed under a microscope LEICA DM2500 with a camera system LEICA type DFC310 FX with Software Leica Application Suite V4.4. Measurements are presented in micrometers, unless indicated, as an average followed by the range (minimum and maximum values) in parentheses. The taxonomic classification of nematodes was performed according to Moravec & Campbell (1991), Moravec et al. (1998), Moravec & Fajer-Avila (2000) and Moravec (2001).

For scanning electron microscopy (SEM), thirty-three nematodes were fixed in 3% Glutaraldehyde, washed in 0.2M phosphate buffer solution, each wash for one hour, post-fixed in 1% osmium tetroxide, dehydrated in progressive alcohol for one hour each (50%, 70%, 80%, 90%, 100%), and dried at the critical point of CO_2_, metallized with gold-palladium and observed in a scanning electron microscope TESCAN model VEGA 3.

Gill fragments with nematodes and eggs were fixed in 10% buffered formalin, and processed for histological analysis, 5 µm thick tissue sections were stained with hematoxylin and eosin (Clarke & Witcomb, 1980; Tolosa et al., 2003). Ecological indices of parasitism were used in accordance with Bush et al. (1997), Bautista-Hernández et al. (2015) and Reiczigel et al. (2019).

## Results

### Search data

A total of 138 nematodes were recovered from the gills of 35 (n=55) specimens of *C. psittacus*, with a prevalence of 63.6%, mean intensity of infection 3.94, mean abundance of 2.50 and range of infection of from 1 to 45 nematodes adults per host. All specimens collected showed characteristics compatible with the genus *Huffmanela* but could not be attributed to a known species; therefore, a new species is described here. The morphological and morphometric characteristics of this new species are presented below and in Table 1.

**Table 1:** Morphology and morphometric comparison of *Huffmanela psittacus* n. sp. collected from *Colomesus psittacus* in Pará, with *Huffmanela* spp. previously published data.

Morphometric characterization	*Huffmanela psittacus* n. sp.		*H. huffmani*		*H. canadensis*		*H. moraveci*		*H. balista*	*H. longa*	
Specimen sex	**Male**	**Female**	Male	Female	Male	Female	Male	Female	Male	Female	Female
Host Locality	***Colomesus psittacus*** **Pará**		*Lepomis* spp., *Amblopites* *rupestris*, *Micropterus salmoides* Texas, USA		*Sebastes* spp. Canada		*Odontesthes smitti*, *O.* *nigricans* Argentina		*Abalistes stellatus* New Caledonia	*Gymnocranius* *grandoculis* New Caledonia	
Total body (TL) ^a^	**3.40–5.60**	**7.50–11.90**	4.69–5.14	4.9–7.51	3.44–3.86	7.71–8.16	3.8–6.4	5–16.3	9.87	4.85	20.73
Maximum body (W) ^b^	**18.30–28.30**	**30–48.30**	24–27	24–30	45–63	90–105	60–90	58–120	30	56	32
cephalic end (W) ^b^	**10.00–16.7**	**15–33.30**	12	12	12	15–18	8–15	10–20	10	16	11
Nerve-ring (L) ^b, c^	**25.00–60.00**	**40–82**	51–57	45–48	48–69	75–78	55–90	70–145	not seen	not seen	not seen
Total esophagus (L) ^a^	**1.00–2.00**	**1.50-2.30**	1.39–1.90	1.29–2.31	1.33–1.34	1.76–2,05	1.15–1.6	1.35–2.35	3.89	1.98	6.72
Muscular esophagus (L) ^a, c^	**65–235**	**190–227**	135–141	150–162	147–153	180–216	110–160	130–260	366	438	181
Muscular esophagus (W) ^b^	**6.7–18.3**	**10–18**	–	–	–	–	–	–		8–9	
Stichosome (L) ^a^	**1.10–1.90**	**1.30–2.10**	1.25–1.76	1.14–2.15	1.18–1,19	1.56–1,83	1–1.5	1.2–2.1	3.52	–	6.51
# Stichocytes	**28–43**	**32–35**	25–26	29–35	36–42	32–37	25–37	30–42	25	–	35
Tail (L) ^b,d^	**3.30–8.30**	**20–73.3**	6–7	–	–		–	–	–	–	–
Vulva (L) ^a,c^	–	**1.50–2.60^c^**	–	0.015–0.030^e^	–	0.039^e^	1.85^c^	1.5–2.8^c^	–	–	0.040^e^
Eggs immature (L × W) ^d×d^	–	**28.3–33.3×16.6–23.3**	–	45×27	–	36-39 × 24-27	–	38–55×23–30	–	41–44 × 17–21	–
Eggs mature (L×W) ^d×d^	–	**36.7–50×18.30–20**	–	60–63×33–39	–	54×26	–	50–57×23–31	–	70.5×34.5	66×26.6
Oesophagus/TL (%)	**30–41%**	**15–27%**	29–41%	26–31%	39%	23%	24–32%	13–21%	39%	–	–
^#^ Specimen	**22**	**10**	10	5	2	3	9	11	1	1	1
Reference	**In this study**		Huffman & Moravec (1988)		Moravec et al. (2005)		Carballo & Navone (2007)		Justine (2007)	Justine (2007)	

Abbreviations: (L) – length; (W) – width; (TL) – full body length. Note: the ranges shown in the table are the variation field in the sample (min – max).

# number;

a Measurements in millimeters unless otherwise indicated;

b Measurements in micrometers (µm);

c Distance from front end;

d Esophageal-intestinal junction;

e End of the esophageal-intestinal junction to the vulva.

Trichinellida Hall, 1916

Trichosomoididae Yorke and Maplestone, 1926

*Huffmanela* Moravec, 1987

**Figure 1:**
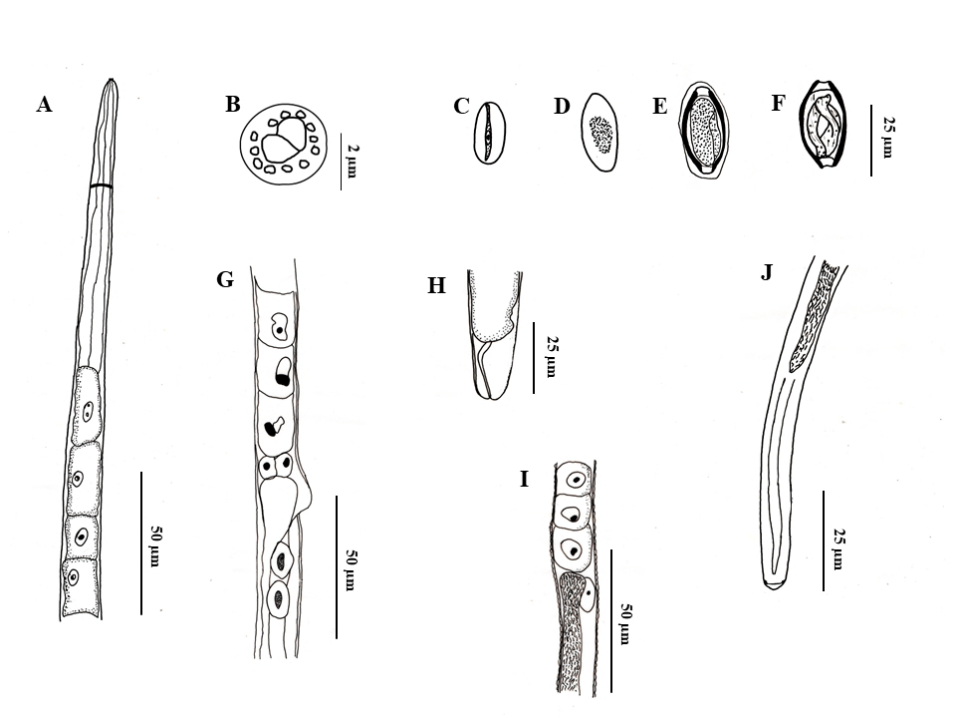
*Huffmanela psittacus* n. sp. in *C. psittacus*. A. Female anterior extremity, lateral view. B. Detail of the female's anterior extremity, prominent lips surrounded by twelve papillae, apical view (reconstructed from SEM micrograph). C. Non-embryonic egg, from inside the uterus of the parasite. D - E. Eggs from gill mucus of developing larval host with envelope covering polar plugs and egg. F. Fully developed egg with larva. G. Stychocytes in the region of the esophageal-intestinal junction and coelomocytes of the female stichosome and vulva, lateral view. H. Female posterior end, side view. I. Esophagus-intestinal junction and male coelomocytes. J. Male posterior end, ventral view.

**Figure 2:**
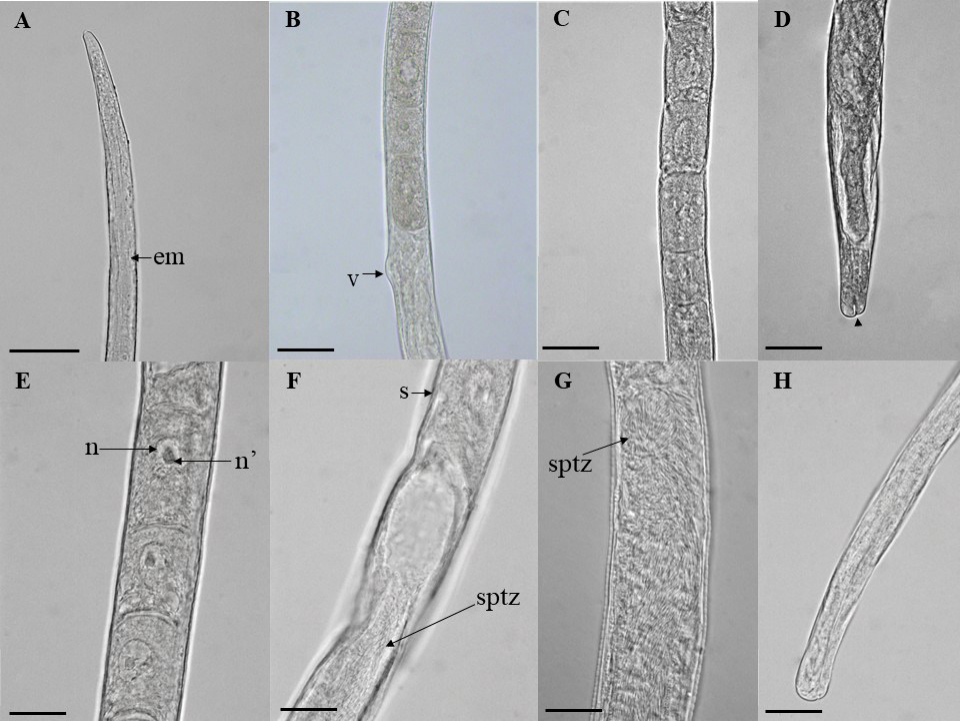
*Huffmanela psittacus* n. sp. in *C. psittacus*. A. Female's anterior extremity, muscular esophagus (em) is observed. Bar: 50µm. B. Esophagus-gut junction with bulge of vulva (v). Bar: 50µm. C. Middle region of the stichosome, showing the stichocytes and large nuclei. Bar: 50µm. D. Female posterior end, anal opening (arrowhead). Bar: 50µm. E. Middle region of male stichosome, nucleus (n) and nucleoli (n'). Bar: 20µm. F - G. Junction esophagus, male intestine, final stichocytes (s), spermatozoa (sptz). Bar: 20µm. H. Male posterior end, tail. Bar: 20µm.

**Figure 3:**
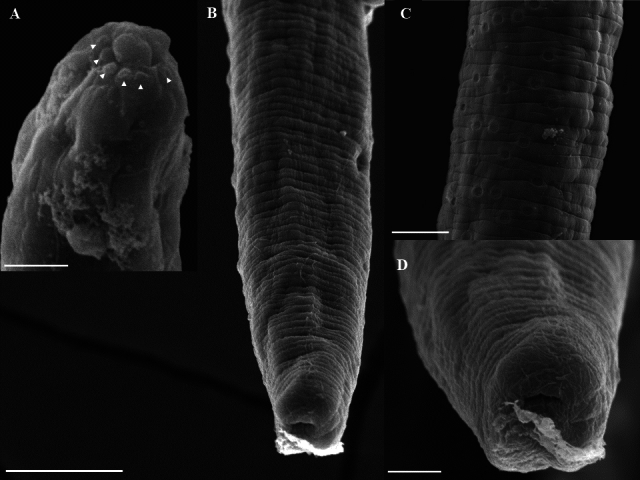
*Huffmanela psittacus* n. sp. in *C. psittacus*. Scanning electron photomicrographs of the male. A. Detail of the male's anterior end, simple lips and some papillae around the lips, apical view (arrowhead). Bar: 2µm. B. Detail of the posterior end of the male, side view. Bar: 20µm. C. Detail of the bacillary bands, lateral view. Bar: 10µm. D. Detail of the posterior end of the male, ventral view. Bar: 5µm.

**Figure 4:**
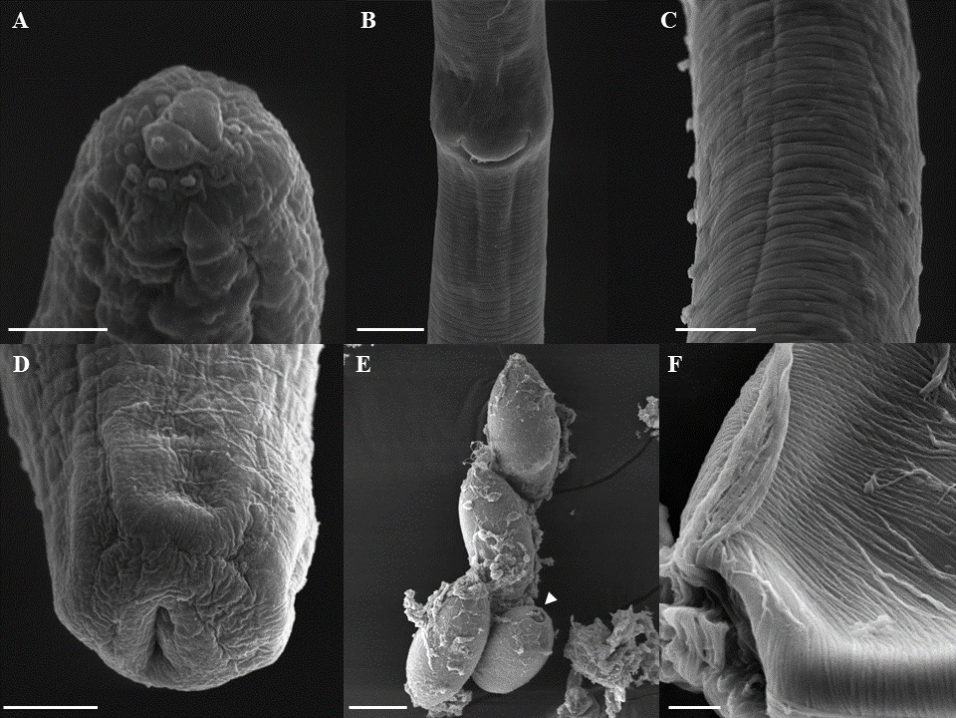
*Huffmanela psittacus* n. sp. in *C. psittacus*. Scanning electron photomicrographs. A. Female anterior end. Bar: 2µm. B. Pre-equatorial region showing the vulva, ventral view. Bar: 20µm. C. Midbody region, ventral and lateral view, showing the bacillary bands. Bar: 10µm. D. Female posterior end, side view. Bar: 5µm. E. Eggs in the mucus of the branchial mucosa, operculated (arrowhead). Bar: 20µm. F. Filamentous eggshell. Bar: 2µm.

*Huffmanela psittacus* n. sp.

(Based on light microscopy and scanning electron microscopy: Figures 1 to 4)

They are small, filiform nematodes, with a narrow anterior end of the body, obtusely rounded, with distinct lips and cephalic papillae (Figure 1B). Cuticle with well-marked transverse striations. Two broad lateral bacillary bands that extend along almost the entire length of the body with numerous more pronounced cuticular projections in males. Narrow and short muscular esophagus and simple posterior stichosome composed of a row of 27−42 stichocytes with large cell nuclei; shorter stichocytes at the back of the stichosome usually subdivided into 2−4 transverse rings; in larger specimens, 1−2 light-colored stichocytes alternating with darker (more granular) stichocytes (Figure 1A; Figure 2C, E). Nerve ring surrounding muscular esophagus in approximately one-third of the posterior area, excretory pore not observed. Small presence of post-esophageal pseudocoelomocytes. Males smaller than females.

**Male (22 specimens, measurements of the holotype in brackets)**Figures 2F-H; Figures 3A-D: Body length 4.70 mm (3.40–5.60) [5,10], maximum width at the esophageal–intestinal junction 24.30 (18.30–28.30) [26.70]; width from cephalic end 13 (10.00–16.7) [14.30], from posterior end 12 (6–15). Total length of esophagus 1.70 mm (1.00–2.00) [1.6], 34.6% (30–41%) [35.30%] of body length, of muscular esophagus 160.40 (65–235) [168.3] × 9.50 (6.7–18.3) [8.30], of stichosome 1.50 mm (1.10–1.90) [1.60]; stichocytes 34 (28–43) [31] in number. Nerve ring 39.90 (25.00–60.00) [40] from anterior end. Single testis reaching anteriorly almost to the esophageal-intestinal junction. Spicule and spicular sheath absent. Caudal end 5.10 (3.30–8.30) [3.30] × 11 (8.30–15) [8.30] slightly narrow, rounded, with pair of large continuous precloacal papillae.

**Female (10 gravid specimens, measurements of allotype in brackets)**: Body length 11.90 mm (7.50–11.90) [10.90], maximum width 39.50 (30–48.30) [40]; width from cephalic end 19 (15–33.30), from posterior end 23.7 (15–30). Length of entire esophagus 2.00 mm (1.50-2.30) [2], 18% (15–27%) [20.70%] of body length, muscular esophagus 205.10 (190–227) [216] × 13.30 (10–18) [13.30], stichosome 1.80 mm (1.30–2.10) [2]; stichocytes 33 (32–35) [34] in number. Nerve ring 67 (40–82) [61.70] of anterior extremity. Vulva situated slightly posterior to esophageal-intestinal junction, 2.10 mm (1.50–2.60) [2.30] from anterior end, anterior vulvar labrum distinctly elevated (Figures 2B, 4B). Short vagina. Eggs oval, light in color, not embryonated, polar plugs of eggs partially developed and not protruding. Eggs and thin-shelled. Eggs arranged in one row in the anterior part of the uterus and in two to three rows posteriorly. Single ovary, extending posteriorly to approximately the level of the end of the intestine. Marked oviduct filled with sperm some distance from the posterior end, terminal anus 51.10 (20–73.3) [56.70] (Figure 4D).

**Eggs inside the uterus:** poorly developed (embryonic), colorless. Outer egg layer with distinct transverse ridges. Non-protruding polar plugs. Eggs 25.10 (28.3–33.3) [31.70] × 14.60 (16.6–23.3) [16.70], wall thickness 0.83 (Figure 1C).

**Eggs in cell division in the gills**: well-developed light-colored embryonated eggs oval, with 2-layer wall; thin light-colored inner layer, thick dark outer layer, with surface bearing numerous complete or incomplete oblique transverse lines (Figure 4E-F). Clearly colored, distinctly protruding polar plugs; polar plug height 5.30 (5–6.70) ×7.70 (6.70–8.30), height of the protruding portion 2–3. Thin clear layer present (envelope) covering the entire egg, including polar plugs (Figure 1E). Egg size, including polar plugs 47 (36.7–50) [50] × 19.30 (18.30–20) [18.30], thick second wall.

**Egg with larvae on gills**: Dark brown eggs containing larvae, oval, with 2-layer wall; thin inner layer and thick dark outer layer, with surface bearing numerous transverse and somewhat complete or incomplete oblique lines (Figure 4E-F). Clearly-colored, distinctly protruding polar plugs; polar plug height 4.80 (2.30–6.70), width 8.70 (8.30–10), height of its protruding portion 2–3. Thin transparent layer present (envelope) covering whole or part of the egg, including polar plugs (Figure 1F). Egg size, including polar plugs 49 (46.7–51.7) [51.33] × 23 (21.7–25) [21.66], thick second wall. Eggs containing fully formed larva.

### Histopathology

**Figure 5:**
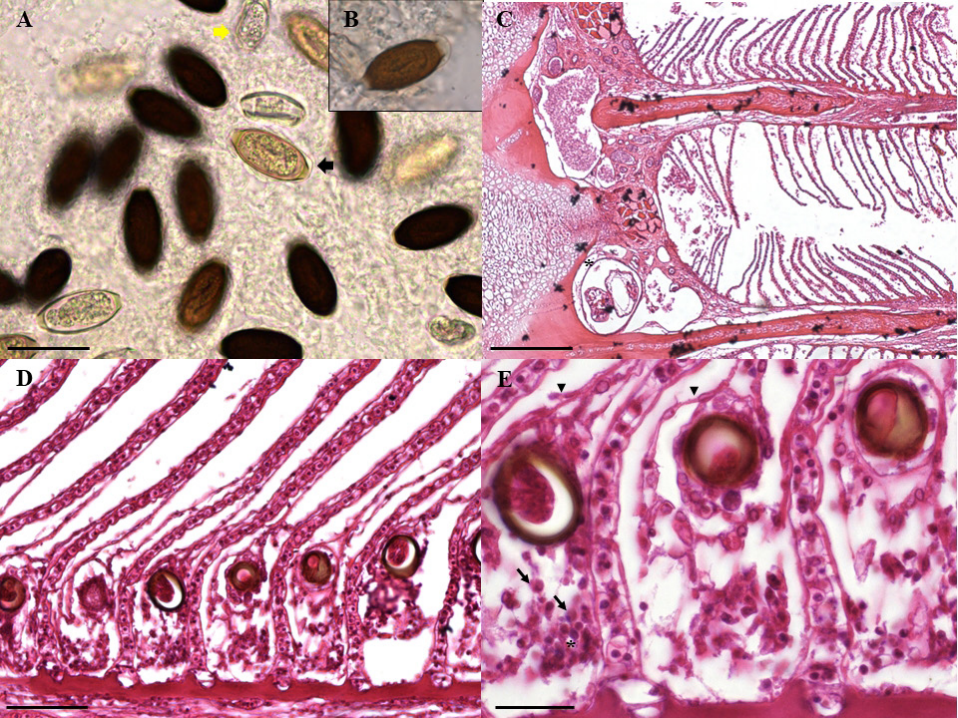
Branchial arch photomicrographs with the presence of *Huffmanela psittacus* n. sp. in *C. psittacus*. Hematoxylin and eosin staining. A. In the mucus of the gill mucosa there are numerous embryonated eggs (yellow arrow) and larvae (black arrow) of *Huffmanela psittacus* n. sp. Bar: 50µm. B. Insert brown larval egg with small amount of clear layer present (envelope) covering entire egg, including polar plugs. Bar=100µm. C. Longitudinal section of the gill arch, with the presence of two nematodes (asterisks). Bar= 200µm. D. Longitudinal section of primary filament, Huffmanela psittacus n. sp. eggs can be seen deposited in an area where there is lamellar fusion. Bar: 50µm. E. The base of the lamella presents edema that promotes detachment of the epithelium (arrowhead) from the secondary lamella. There is also a special granulocytic cell (arrows) and lymphocytic infiltrate (asterisk) at the base of the gill filament. Bar=20µm.

Fresh tissue specimens examined using light microscopy showed multiple rounded to oval dark eggs (at different developmental stages) along with larvae and adults. On histological section, the gill arch, lamellae, and filaments showed numerous mature and larval eggs of the parasite dispersed in the gill mucus. The base of the lamella presents edema that promotes detachment of the epithelium from the secondary lamella. There is also a special granulocytic cell and lymphocytic infiltrate at the base of the gill filament (Figure 5).

### Taxonomic summary

**Host**: *Colomesus psittacus* (Bloch & Schneider, 1801) (Tetraodontiformes: Tetraodontidae).

**Common name**: pufferfish, banded pufferfish, parrot puffer.

**Site of infection**: gill arch, gills and gill-mucosa.

**Biome**: Amazon.

**Location**: municipality of Soure (00º 43' 00” S; 48º 31' 24” W), Marajó Island, State of Pará.

**Etymology**: The specific name *psittacus* (genitive) relates to the specific name of the host fish (*psittacus*).

**Specimen deposit**: Holotype (CHIOC 39324a), allotype (CHIOC 39324b) and paratypes were deposited in the Coleção Helmintológica do Instituto Oswaldo Cruz (CHIOC), Rio de Janeiro, Brazil: 4 males (CHIOC 39324c), (CHIOC 39324d), (CHIOC 39324e), (CHIOC 39324f) and 4 females (CHIOC 39324g), (CHIOC 39324h), (CHIOC 39324i), (CHIOC 39324j).

## Discussion

The nematodes of the genus *Huffmanela* described so far are parasites of fish, with the main sites of infection being the skin, oral cavity, musculature, swim bladder, gills and serosa of the intestines (Bullard et al., 2012; Carballo & Navone 2007; Esteves et al., 2009, 2016; Huffman & Moravec, 1988; Moravec & Garibaldi, 2000; Ruiz & Bullard, 2013). In the gills, the record of infections by nematodes in fish is by the larval stage of *Contracaecum* sp. in *Oligoplites palometa*, *O. saliens*, and *O. saurus*, and the adult form of *Raphidascaris* sp. in *Pinguipes brasilianus* from Brazil by Luque et al. (2011). There are still few studies about the parasitic helminths of *C. psittacus*, mainly referring to the gills.

The parasites present in the gill mucosa of *C. psittacus*, in this study, have similar characteristics to those of the Trichosomoididae family. This family includes a genus *Huffmanela* with the following species described in fish, based on the eggs of these parasites: *H. banningi* Moravec, 1987 in the musculature of Cynoglossidae fish in Africa; *H. carcharhini* (MacCallum, 1925) on the skin and gills of Carcharhinidae in the United States; *Huffmanela* sp. (Grabda & Ślósarczyk, 1981) Moravec, 2001 on Ophidiidae in the Pacific Ocean, New Zealand and the South Island; *H. schouteni* Moravec & Campbell, 1991 in the swim bladder of Exocoetidae in Italy; *H. japonica* Moravec et al. 1998 in the musculature of Mullidae in Japan; *H. shikokuensis* Moravec et al. 1998 in the musculature of Monacanthidae in Japan; *H. mexicana* Moravec & Fajer-Avila, 2000 in the swim bladder of Tetraodontidae (*Sphoeroides annulatus*) in Mexico; *H. paronai* Moravec & Garibaldi, 2000 in Xiphiidae on skin in Italy; *H. branchialis* Justine, 2004 in Nemipteridae on gill bones and arch in New Caledonia; *H. filamentosa* Justine, 2004 in Lethrinidae in New Caledonia; *H. ossicola* Justine, 2004 in the gills of Labridae in New Caledonia; *H. lata* Justine, 2005 in Carcharhinidae on skin in New Caledonia; *H. plectropomi* Justine, 2011 in the mesentery of Serranidae in New Caledonia; *H. oleumimica* Ruiz & Bullard, 2013 in the skin, gills and oral cavity of Lutjanidae in the Gulf of Mexico; *H. markgracei* Ruiz & Bullard, 2013 in Carcharhinidae on tongue skin, gill arches and posterodorsal vestibular cavity in the Gulf of Mexico; *H. hamo* Justine & Iwaki, 2014 in the musculature of Muraenesocidae in Japan, and *H. lusitana* Ramos et al., 2019 in the epaxial and hypaxial musculature off the Atlantic coast of Portugal.

According to Moravec (1987, 2001) and Gibbons (2010), they are small and filamentous nematodes, with a stichosome composed of a single row of stichocytes with presence of post-esophageal glands. Relatively long cloaca in the male, spicule and spicule sheath absent. Posterior extremity of male with dorsoventral depression, without membranous bursa, provided with a pair of adcloacal papillae. Terminal female anus, oviparous, with vulva near the end of the esophagus. Non-embryonic eggs in the uterus; eggs laid in host tissues. Egg with larvae have heavily pigmented, dark walls, often noticeably thick; whole egg including polar plugs embedded in a thin transparent envelope usually densely covered by tiny spines. The type species is *H. carcharhini* (MacCallum, 1925).

Adult specimens recorded in fish are: *H. huffmani* Moravec, 1987 in the swim bladder in Centrarchidae in Texas, United States; *H. canadensis* Moravec, Conboy & Speare, 2005 on the skin of the fins and adjacent to the fins of Sebastidae in Canada; *H. ballista* Justine, 2007 in the external mucosa of the swim bladder in Balistidae; *H. longa* Justine, 2007 in the mesentery, external mucosa of the swim vesicle and mucosa of the dorsal wall in Lethrinidae in New Caledonia; *H. moraveci* Carballo and Navone, 2007 in Atherinopsidae on pectoral and anal fin skin, operculum epithelium, gill mucosa in Argentina; *Huffmanela* sp. Al-Hasson et al. 2019 in the mesentery of Sparidae in Iraq. The nematodes in this study showed morphological and morphometric characteristics compatible with *Huffmanela*, but the adults differ from those previously recorded.

Identification of *Huffmanela* species is based on keys created by egg characteristics such as size and shell surface morphology (Moravec & Campbell, 1991; Moravec et al., 1998; Moravec & Fajer-Avila, 2000; Moravec, 2001; Conboy & Speare, 2002). According to Moravec & Campbell (1991), Moravec & Garibaldi (2003)*H. schouteni* has a thin envelope with bulges; Moravec et al. (1998)*H. japonica* has a thin and smooth envelope, with the presence of protuberances only in developing eggs, and *H. shikokuensis* smooth surface of the egg; Justine (2007)*H. longa* has elongated stichocytes and eggs with longitudinal folds, *H. ballista* egg has a surface marked by imperceptible longitudinal ridges, and *H. ossicola* has a characteristic egg envelope and numerous filaments. Justine (2011) observed that in *H. plectropomi* the surface of the eggs has a thick, continuous layer of filaments, but no envelope around the egg. Justine & Iwaki (2014) observed that the eggs of *H. hamo* had a smooth surface and did not have an envelope or filaments, and the surface of *H. lusitana* eggs do not have spines or filamentous structures (Ramos et al., 2019).

Species with the presence of surface filaments in their eggs, similar to our study, include *H. ossicola* Justine, 2004; *H. filamentosa* Justine, 2004; *H. canadensis* Moravec, Conboy & Speare, 2005; *H. longa* Justine, 2007 (Justine, 2004; 2007; Moravec & Fajer-Avila, 2000), and *H. plectropomi* Justine, 2011; *H. markgracei* Ramos et al., 2019 and *Huffmanela* sp. Attia et al. 2021a (Table 2). When analyzing the morphology and morphology of the eggs with the other species, we can observe that the eggs in our study are smaller, and do not match those of any of the recorded species and using SEM we observed the presence of very marked filaments on the surface of the eggshell. Larval egg.

**Table 2:** Morphology and morphometric comparison of eggs *Huffmanela psittacus* n. sp. collected from *Colomesus psittacus* in Pará, with *Huffmanela* spp. other hosts of world.

Morphometric characterization		*Huffmanela psittacus* n. sp.						*H. huffmani*					*H. schouteni*					*H. japonica*						*H. shikokuensis*		*H. paronai*
Host Locality		***Colomesus psittacus* Pará**						*Lepomis* spp., *Amblopites rupestris*, *Micropterus salmoides* Texas, USA					*Hirundichthys affinis, Cypselurus* Netherlands antilles					*Upeneus bensasi*; *Stephanolepis cirrhifer* Japan						*Stephanolepis* spp. Japan		*Xiphias gladius* Western Mediterranean
										
Eggs immature (L×W) ^a^		**25.1–33.3×16.6–23.3**						45×27					–					46×25						–		43–50×19–23
Eggs mature (L×W) ^a^		**46.7–51.7×21.7–25**						60–63×33–39					69-75 × 27-30					58-69×26-30						78–90×36-45		48–51 × 21–24
Shell surface morphology		**Longitudinal, oblique and transverse ridges**						spinose					thin envelope with protuberances					spinose						grainy		smooth surface
Site of infection		**gill arch, gills and gill-mucosa**						swimbladder					serosa of intestine, abdominal cavity, swimbladde					muscle						muscle		Epidermis^b^
Reference		**In this study**						Huffman & Moravec (1988)					Moravec & Campbell (1991)					Moravec et al. (1998)						Moravec et al. (1998)		Moravec & Garibaldi (2000)
		***Huffmanela psittacus* n. sp.**						*H. mexicana*					*H. banningi*					*H. carcharini*						*Huffmanela* spp.		*H. schouteni*
Host Locality		***Colomesus psittacus* Pará**						*Sphoeroides annulatus* Mexico					*Cynoglossus browni* Atlantic Ocean, Senegal and Congo					*Carcharhinus melanopterus, C. plumbeus* Atlantic Ocean, USA						*Sebastes* spp. Canada		*Cheilopogon heterurus* Europe
										
Eggs immature (L × W) ^a^		**25.1–33.3×16.6–23.3**						–					–					–						–		–
Eggs mature (L×W) ^a^		**46.7–51.7×21.7–25**						63–69×30–33					99–108×42–45					90–105 × 42–54						50–55×21–27		69–75×30–33
Shell surface morphology		**Longitudinal, oblique and transverse ridges**						smooth surface					spinose					envelope visible at pole regions						Tranverse ridges		–
Site of infection		**gill arch, gills and gill-mucosa**						swimbladder					muscle					skins and mucosa of connective tissue of gill arches						skin		swimbladder
Reference		**In this study**						Moravec & Fajer-Avila (2000)					Moravec (2001)					Moravec (2001)						Conboy & Speare (2002)		Moravec & Garibaldi (2003)
						
Morphometric characterization	*Huffmanela psittacus* n. sp.						*H. filamentosa*					*H. branchialis*					*H. ossicola*					*Huffmanela* sp.			*H. lata*	
Host Locality	***Colomesus psittacus* Pará**						*Gymnocranius grandoculis* New Caledonia					*Nemipterus furcosus* New Caledonia					*Bodianus loxozonus B. perditio, B. busellatus* New Caledonia					*Pentapodus aureofasciatus* New Caledonia			*Carcharhinus amblyrhynchos* Pacific Ocean, New Caledonia	
										
Eggs immature (L × W) ^a^	**25.1–33.3×16.6–23.3**						–					–					–					–			–	
Eggs mature (L×W) ^a^	**46.7–51.7×21.7–25**						48–53×25–30					45–52×23–30					72–88 × 32–40					39–47 × 22–27			77–88 × 52–63	
Shell surface morphology	**Longitudinal, oblique and transverse ridges**						filaments at extremities					aspinose					numerous filaments on eggs surrounded by thin envelope					no filaments			spinose	
Site of infection	**gill arch, gills and gill-mucosa**						gill–mucosa					gills-filaments					within all bones, including gill arch bones					mucosa of gills			skin	
Reference	**In this study**						Justine (2004)					Justine (2004)					Justine (2004)					Justine (2004)			Justine (2005)	

	***Huffmanela psittacus* n. sp.**						*Huffmanela* sp.					*H. canadensis*					*H. moraveci*					*H. balista*			*H. longa*	
Host Locality	***Colomesus psittacus* Pará**						*Carcharhinus plumbeus* North Carolina, USA					*Sebastes* spp. Canada					*Odontesthes smitti*, *O. nigricans* Argentina					*Abalistes stellatus* New Caledonia			*Gymnocranius grandoculis* New Caledonia	
										
Eggs immature (L × W) ^a^	**25.1–33.3×16.6–23.3**						–					36–39 × 24–27					26–44×21–28					–			–	
Eggs mature (L×W) ^a^	**46.7–51.7×21.7–25**						73–86 × 39–47					48–63 × 26 24–27					50–57×23–31					63–78 × 32–41			58–69 × 26–34	
Shell surface morphology	**Longitudinal, oblique and transverse ridges**						–					numerous transverse and somewhat oblique, complete or incomplete ridges					lightly ornamented					no filament			long thin filaments at eggs extremities	
Site of infection	**gill arch, gills and gill-mucosa**						skin					skin					skin and gills					outer swimbladder wall			mesentery, mucosa of abdominal cavity, outer Swimbladder wall	
Reference	**In this study**						MacLean et al. (2006)					Moravec et al. (2005)					Carballo & Navone (2007)					Justine (2007)			Justine (2007)	
Morphometric characterization	*Huffmanela psittacus* n. sp.					*Huffmanela* sp.						*H. plectropomi*					*H. carcharhini*						*H. oleumimica*		*H. markgracei*	
Host Locality	***Colomesus psittacus* Pará**					*Trisopterus luscus* Portuguese coast						Plectropomus leopardus New Caledonia					*Carcharhinus plumbeus* Pacific Ocean						*Lutjanus campechanus* Northern Gulf of Mexico		*Rhizoprionodon terraenovae* Gulf of Mexico	
										
Eggs immature (L × W) ^a^	**25.1–33.3×16.6–23.3**					33–57×26–38						–					–						47–53×26–32		97–109×40–44	
Eggs mature (L×W) ^a^	**46.7–51.7×21.7–25**					73–94×39–59						64–76×29–35					75–95×48–63						160–201×7–8		90–113×39–54 99–109×39-49	
Shell surface morphology	**Longitudinal, oblique and transverse ridges**					–						filaments					spinose						spinose		transverse ridges	
Site of infection	**gill arch, gills and gill-mucosa**					Muscle, skin, mucosa intestine, swimbladder						mesentery					skin						epidermis of urohyal, branchiostegals, and buccal cavity		skin of tongue, branchial arches and buccal cavity	
Reference	**In this study**					Esteves et al. (2009)						Justine (2011)					Bullard et al. (2012)						Ruiz et al. (2013)		Ruiz & Bullard (2013)	

	***Huffmanela psittacus* n. sp.**					*H. hamo*						*Huffmanela* spp.					*H. lusitana*						*Huffmanela* sp.		*Huffmanela sp.*	
Host Locality	***Colomesus psittacus* Pará**					*Muraenesox cinereus* Japan						*Microchirus azevia* Coast atlantic					*Trisopterus luscus* Atlantic coast of Portugal						*Carcharhinus dussumieri* Saudi Arabia		*Epinephelus coioides* Saudi Arabia	
										
Eggs immature (L × W) **^a^**	**25.1–33.3×16.6–23.3**					66–77×33–38						47–51×26–32					39.7–59.1×22–36.8						92–110×35–48		60.3–75.6×26.4–36.5	
Eggs mature (L×W) ^a^	**46.7–51.7×21.7–25**					70–79×34–39						75–86×37–42					69.1–82.9×36.9–45.7						95–107×34–49		–	
Shell surface morphology	**Longitudinal, oblique and transverse ridges**					smooth						–					smooth						filaments or ridges		–	
Site of infection	**gill arch, gills and gill-mucosa**					muscle						Muscle, gill arch, skin					Epaxial and hypaxial musculature						skin		muscle	
Reference	**In this study**					Justine & Iwaki (2014)						Esteves et al. (2016)					Ramos et al. (2019)						Attia et al. (2021a)		Attia et al. (2021b)	

Abbreviations: (L) – length; (W) – width.

a Measurements in micrometers, size of clear eggs including polar plugs;

b Mainly on lower jaw, gill covers, pectoral, anal and caudal fins and lower half of body.

The adult nematodes recorded so far are few, as shown in Table 1, and very well characterized through scientific illustration and morphological description of the adults (Moravec et al., 2005; Carballo & Navone, 2007; Justine, 2007; Al-Hasson et al., 2019). Using scientific illustrations and SEM Carballo & Navone (2007) satisfactorily characterized the species *H. moraveci*, a parasite of Atherinopsidae fish. Thus, it was possible to distinguish the species of this study, as in its anterior region at the cephalic end it has an oral opening with a circular shape, and simple lips surrounded by a rosette with twelve papillae. In addition, there are two bands of lateral bacillary bands. Therefore, due to the morphological and morphometric differences of the adults and eggs of the nematodes in our research, we can identify a new species, which we have called *Huffmanela psittacus* n. sp. a parasite of *C. psittacus* in Marajó Bay, Pará State, Brazil.

In this study *Huffmanela psittacus* n. sp. adults and eggs were observed only in the gill arch and gills of the host, which triggered mild lymphocytic infiltration of the epithelium between the secondary lamellae present. In *Sebastes* spp. Conboy & Speare (2002) observed epithelial hyperplasia, spongiosis and mild lymphocytic infiltration of the epithelium and underlying dermis due to the presence of eggs in the gills. In *Trisopterus luscus*Esteves et al. (2009) observed in myodegeneration, necrosis, calcification and small granulomas with parasites; fish with hyperinfection showed diffuse histiocytic infiltrate and fibrosis, while fish skin, intestinal serosa and swim bladder showed no abnormalities; Bullard et al. (2012) observed intraepithelial inflammation with eosinophilic granulocytes and hyperplasia as well as lymphofollicular hyperplasia in the dermis of *Carcharhinus plumbeus*.

Eissa et al. (2021) observed hemorrhagic, necrotic, and chronic inflammatory cell aggregations in the tissue surrounding the swim bladder, as well as tunnels of necrotic tissue that contained the eggs; Attia et al. (2021b) observed an intense granulomatous reaction with infiltration of mononuclear inflammatory cells and proliferation of fibrous connective tissue in the histopathology of the muscle fibers of *Epinephelus coioides* containing the nematodes and eggs. In the histological analysis of the gills of *C. psittacus*, in this study, numerous eggs were observed between the lamellae, which may have caused hyperplasia of the squamous epithelium of the primary and secondary filaments. Changes in fish gills can have several causes, such as the presence of parasites and/or environmental changes (De Lima et al., 2009; Santos et al., 2014).

## Conclusions

Based on the differences mentioned, we can therefore say that the nematodes in this research are a new species, we call named *Huffmanela psittacus* n. sp. This finding represents the first record of a species of *Huffmanela* in South America and the first record in the gills of a Tetraodontiformes fish, *C. psittacus* in the state of Pará. We have also added the description of histopathological changes caused by this parasite, thus contributing to the knowledge of its pathogenicity in *C. psittacus* on Marajó Island.
